# Developmental Trajectories and Predictors of Incident Dementia among Elderly Taiwanese People: A 14-Year Longitudinal Study

**DOI:** 10.3390/ijerph20043065

**Published:** 2023-02-09

**Authors:** Yen-Chun Fan, Sheng-Feng Lin, Chia-Chi Chou, Chyi-Huey Bai

**Affiliations:** 1 School of Public Health, College of Public Health, Taipei Medical University, Taipei 110301, Taiwan; 2 Department of Public Health, School of Medicine, College of Medicine, Taipei Medical University, Taipei 110301, Taiwan; 3 Department of Emergency Medicine, Taipei Medical University Hospital, Taipei 110301, Taiwan; 4 Institute of Epidemiology and Preventive Medicine, National Taiwan University, Taipei 100025, Taiwan; 5 School of Medicine, Chang Gung University, Taoyuan 33302, Taiwan; 6 Department of Internal Medicine, Chang Gung Memorial Hospital, Keelung 204201, Taiwan; 7 Nutrition Research Center, Taipei Medical University Hospital, Taipei 110301, Taiwan

**Keywords:** trajectory, group-based trajectory model, predictors, dementia, longitudinal

## Abstract

The aim of this study was to identify dementia trajectories and their associated predictors among elderly Taiwanese people over a 14-year period using a nationwide representative longitudinal study. This retrospective cohort study was performed using the National Health Insurance Research Database. Group-based trajectory modeling (GBTM) was used to distinguish the specific trajectory groups of incident dementia during 2000–2013. All 42,407 patients were classified by GBTM to identify the trajectory of incident dementia, which included high- (*n* = 11,637, 29.0%), moderate- (*n* = 19,036, 44.9%), and low-incidence (*n* = 11,734, 26.1%) groups. Those diagnosed with hypertension (adjusted odds ratio [aOR] = 1.43; 95% confidence interval [CI] = 1.35–1.52), stroke (aOR = 1.45, 95% CI = 1.31–1.60), coronary heart disease (aOR = 1.29, 95% CI = 1.19–1.39), heart failure (aOR = 1.62, 95% CI = 1.36–1.93), and chronic obstructive pulmonary disease (aOR = 1.10, 95% CI = 1.02–1.18) at baseline revealed tendencies to be classified into high-incidence groups in dementia risk. The results from a 14-year longitudinal study identified three distinct trajectories of incident dementia among elderly Taiwanese people: patients with cardiovascular disease risk factors and cardiovascular disease events tended to be classified into high-incidence dementia groups. Early detection and management of these associated risk factors in the elderly may prevent or delay the deterioration of cognitive decline.

## 1. Introduction

Despite the age-adjusted incidence rate of dementia being relatively stable worldwide, the total number of people living with dementia has more than doubled over the past two decades [1]. Approximately 153 million dementia cases have been forecasted in the next 30 years, particularly within the aging population in East Asia [2]. The burden of Alzheimer’s disease (AD) and dementia has increased with their rising incidence, prevalence, and mortality [3,4]. It is estimated that dementia will be the most costly disease and a major public health issue for unpaid caregivers [5].

Several predictors independently associated with the risk of dementia, such as cardiovascular disease events [6], cardiovascular disease risk factors [7], and psychological disorders [8], have been demonstrated. The management of midlife and later-life modifiable risk factors for dementia might potentially prevent approximately 40% of dementia cases [9]. Moreover, the reduced incidence of dementia could benefit from intensive interventions and changes to lifestyle in high-risk populations presenting with mild cognitive impairment (MCI) [10]. The causes of dementia vary based on the changing pattern of dementia progression, and numerous commonly recognized risk factors have been identified [11]. Therefore, it is necessary to identify factors associated with dementia severity.

Previous studies have used group-based trajectory modeling (GBTM) to identify the longitudinal trajectories for cognitive impairment, while heterogeneous patterns in the temporal trend of dementia have been presented due to inconsistencies in the definition of dementia, data source, duration of follow-up, and cognitive assessment [12,13,14,15]. To the best of our knowledge, no study has examined long-term patterns using a nationwide representative sample of the Taiwanese population. Moreover, GBTM can be applied to depict the dynamic trajectories throughout dementia progression, which provides group members with similar characteristics [16,17]. Thus, it may help distinguish specific patterns across disease states.

This study aimed to identify dementia trajectories among elderly Taiwanese people over a 14-year period using a nationwide representative longitudinal study. Whether demographic characteristics and baseline comorbidities were associated with the trajectory groups was further examined. Moreover, a sensitivity analysis was performed for the whole study sample to cross-validate the relationship between baseline factors and the risk of developing dementia and to examine the differences in dementia risk among the three distinct trajectory groups.

## 2. Materials and Methods

### 2.1. Data Sources

A retrospective cohort study design was used in this study. The data were obtained from the National Health Insurance Research Database (NHIRD), which covers 99.9% of residents in Taiwan. The study participants were retrieved from the Longitudinal Health Insurance Database 2010 (LHID 2010), a subset of the NHIRD. LHID 2010 contained one million beneficiaries randomly sampled from the Registry for Beneficiaries in 2010. It includes claims data regarding ambulatory visits, inpatient admissions, and prescription records. Moreover, there were no significant differences in the distribution of age, sex, and average insurance amount between LHID 2010 and the NHIRD. To protect personal privacy, all researchers must sign a written agreement to declare that they have no intention of obtaining information when using the NHIRD and its data subsets. Diseases were diagnosed based on the International Classification of Diseases, Ninth Revision, Clinical Modification (ICD-9-CM) codes. The accuracy of the diagnostic diseases in the NHIRD has been validated [18,19]. This study was approved and reviewed by the Joint Institutional Review Board of Taipei Medical University (TMU-JIRB No.: N201606010).

### 2.2. Study Population

Participants who had any ambulatory visit or hospital admission were identified from 1 January 2000 to 31 December 2000. The index date was defined as the date of the first outpatient or inpatient visit in 2000. Those who had either a primary or secondary diagnosis of dementia before the index date were excluded to investigate the trajectory of incident dementia. In addition, at the 2000 baseline, individuals aged less than 65 years or greater than 99 years and subjects with missing sex information were excluded. Ultimately, 42,407 patients were enrolled in the final analysis.

### 2.3. Identification of Dementia

All individuals in this study were tracked from the index date to the end of 2013 to identify the subsequent development of dementia. Moreover, annual data of those with new-onset dementia annually during the 14-year trajectory period (2000–2013) were retrieved, which could be used in group-based trajectory models. In addition, all subjects were grouped according to the dichotomous status of new-onset dementia. Therefore, there were no missing values in the sample. Dementia events were defined based on either a primary or secondary ICD-9-CM diagnosis code, with at least three outpatient visits or at least one inpatient visits, including senile dementia, uncomplicated (290.0), presenile dementia (290.1×), senile dementia with delusional or depressive features (290.2×), senile dementia with delirium (290.3), arteriosclerotic dementia (290.4×), dementia in conditions classified elsewhere (294.1), Alzheimer’s disease (331.0), Pick’s disease (331.1), and senile degeneration of the brain (331.2) [20].

### 2.4. Covariates

To examine the factors associated with incident dementia, several covariates at baseline were considered and adjusted in the regression models. Baseline information on demographic characteristics regarding the age and sex of eligible individuals was gathered from claims data at the index date. The comorbid conditions were obtained within 1 year before the index date, which included either a primary or secondary diagnosis of diabetes mellitus (ICD-9-CM 250), hypertension (ICD-9-CM 401–405), hyperlipidemia (ICD-9-CM 272), stroke (ICD-9-CM 430–438), coronary heart disease (CHD; ICD-9-CM 410–414), kidney disease (ICD-9-CM 580–589), atrial fibrillation (ICD-9-CM 427.3), depression (ICD-9-CM 296.2–296.3, 300.4, and 311), anxiety (ICD-9-CM 300.0, 300.2–300.3, 308.3, and 309.81), heart failure (ICD-9-CM 428), alcoholism (ICD-9-CM 303 and 305.0), chronic obstructive pulmonary disease (COPD; ICD-9-CM 490–496), and obesity (ICD-9-CM 278, 278.0, 278.00, and 278.01).

### 2.5. Statistical Analysis

A group-based trajectory modeling using zero-inflated Poisson (ZIP) mode was applied to distinguish the specific trajectory groups of the incident dementia rate over a 14-year period (2000–2013). GBTM is an application of a finite mixture, which assumes that it is composed of a mixture of distinct groups within the population, identified by developmental trajectories [21]. The SAS procedure of PROC TRAJ was used to analyze GBTM for the clustering of the longitudinal data. The PROC TRAJ macro allowed us to handle the maximum likelihood estimates to fit the nonlinear model [16]. Each model was tested and fitted to data with linear, quadratic, and cubic functions for the trajectories of dementia with 2–6 groups. In addition, the demographic variables of age and sex, which are unmodifiable factors affecting the probability of group membership, were also included in GBTM to identify the distinct trajectories sharing similar patterns of demographic factors. The best model was chosen based on the lowest Bayesian information criterion value to determine the optimal number of groups for the incidence of dementia over time.

Continuous variables are presented as mean and standard deviation. Dichotomous variables are expressed as numbers and percentages. The Kruskal–Wallis H test or the chi-square test were used to examine the difference in distribution of baseline sample characteristics and comorbid conditions between the dementia trajectory groups. Moreover, a multinomial logistic regression was used to estimate the odds ratios (ORs) and 95% confidence intervals (CIs) with adjustment for diabetes mellitus, hypertension, hyperlipidemia, stroke, coronary heart disease, kidney disease, atrial fibrillation, depression, anxiety, heart failure, alcoholism, chronic obstructive pulmonary disease, and obesity to explore the association between baseline characteristics and dementia trajectory groups. In addition, a sensitivity analysis was conducted to cross-validate the study results by: (1) implementing multivariate Cox proportional hazard regressions to investigate the predictors of dementia for the whole sample; and (2) using the Kaplan–Meier method with a log-rank test to examine differences in dementia risk among three distinct trajectory groups. All statistical analyses were performed using SAS version 9.4 for Windows (SAS Institute Inc., Cary, NC, USA), and the significance level was set at a *p* value less than 0.05.

## 3. Results

### 3.1. Developmental Trajectories and Its Baseline Demographic Characteristics

**Figure 1:**
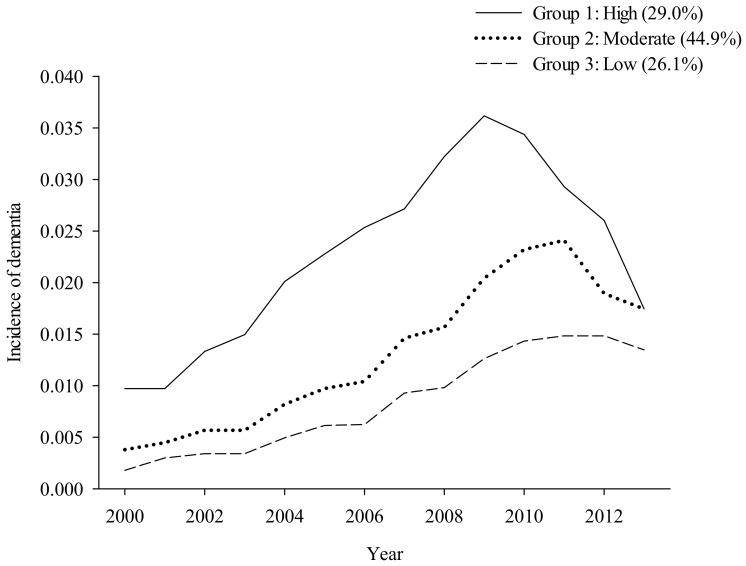
Trajectory of dementia incidence between 2000–2013. Group 1: high dementia incidence (29.0% probability); Group 2: moderate dementia incidence (44.9% probability); Group 3: low dementia incidence (26.1% probability).

All 42,407 patients were classified using GBTM to identify the trajectory of incident dementia. Three trajectory groups were identified between 2000 and 2013: group 1 with a high dementia incidence (*n* = 11,637, 29.0% probability), group 2 with a moderate dementia incidence (*n* = 19,036, 44.9% probability), and group 3 with a low dementia incidence (*n* = 11,734, 26.1% probability; Figure 1).

As shown in Table 1, the distribution of sample characteristics among the dementia trajectory groups significantly differed in mean age (*p* < 0.001), the percentage of women (*p* < 0.001), and the prevalence of diabetes mellitus (*p* < 0.001), hypertension (*p* < 0.001), hyperlipidemia (*p* = 0.012), stroke (*p* < 0.001), coronary heart disease (*p* < 0.001), kidney disease (*p* = 0.004), atrial fibrillation (*p* = 0.005), anxiety (*p* = 0.009), heart failure (*p* < 0.001), chronic obstructive pulmonary disease (*p* < 0.001), and obesity (*p* = 0.024). The groups did not significantly differ in the prevalence of depression (*p* = 0.387) or alcoholism (*p* = 0.462).

### 3.2. Factors Associated with the Trajectory Groups

The multivariate multinomial logistic regression results showed that hypertension (adjusted OR [aOR] = 1.43, 95% CI = 1.35–1.52, *p* < 0.001), stroke (aOR = 1.45, 95% CI = 1.31–1.60, *p* < 0.001), coronary heart disease (aOR = 1.29, 95% CI = 1.19–1.39, *p* < 0.001), heart failure (aOR = 1.62, 95% CI = 1.36–1.93, *p* < 0.001), and chronic obstructive pulmonary disease (aOR = 1.10, 95% CI = 1.02–1.18, *p* = 0.009) at baseline were positively associated with a high dementia incidence compared with a low dementia incidence, whereas hyperlipidemia (aOR = 0.81, 95% CI = 0.74–0.89, *p* < 0.001) was negatively associated (Table 2). In addition, similar results were found for patients with diabetes mellitus (aOR = 1.08, 95% CI = 1.00–1.16, *p* = 0.039), hypertension (aOR = 1.27, 95% CI = 1.20–1.34, *p* < 0.001), stroke (aOR = 1.14, 95% CI = 1.03–1.25, *p* = 0.008), coronary heart disease (aOR = 1.16, 95% CI = 1.07–1.24, *p* < 0.001), heart failure (aOR = 1.19, 95% CI = 1.00–1.41, *p* = 0.048), chronic obstructive pulmonary disease (aOR = 1.11, 95% CI = 1.04–1.19, *p* = 0.002), and obesity (aOR = 2.50, 95% CI = 1.03–6.06, *p* = 0.043), with higher odds of dementia incidence in the moderate group than in the low group.

### 3.3. Sensitivity Analysis

**Figure 2:**
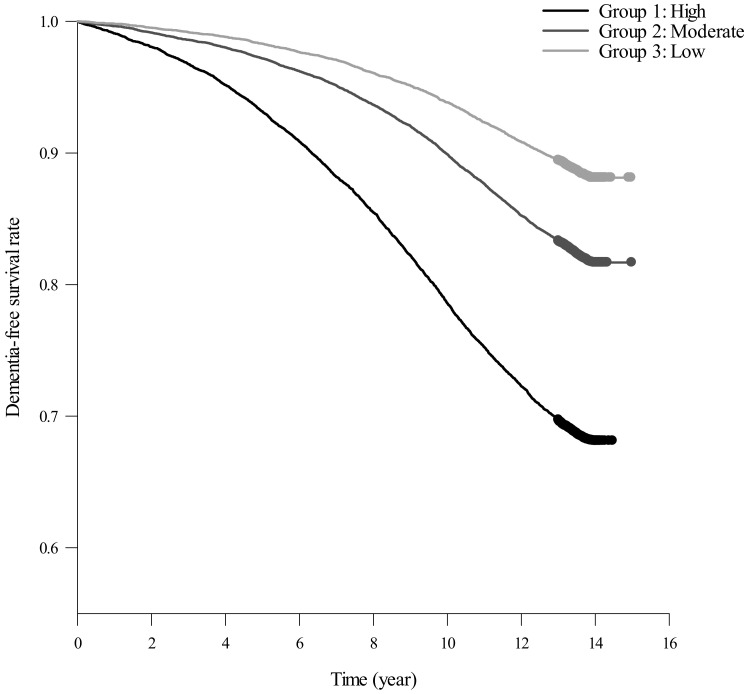
Dementia-free survival rate among three distinct trajectory groups in dementia incidence as estimated by Kaplan–Meier method (Log-rank test: *p* < 0.001; all pairwise comparisons: *p* < 0.001).

Among the 42,407 participants, 8566 developed dementia during 543,047 person-years in up to 14 years of follow-up. Table 3 presents the baseline characteristics associated with the risk of dementia for the entire sample using multivariate Cox proportional hazard regression. Several crucial risk factors for the development of dementia were found, including the baseline factors of age (adjusted HR [aHR] = 1.08, 95% CI = 1.08–1.09, *p* < 0.001), female sex (aHR = 1.20, 95% CI = 1.15–1.25, *p* < 0.001), diabetes mellitus (aHR = 1.26, 95% CI = 1.18–1.34, *p* < 0.001), stroke (aHR = 1.61, 95% CI = 1.51–1.72, *p* < 0.001), coronary heart disease (aHR = 1.13, 95% CI = 1.06–1.20, *p* < 0.001), kidney disease (aHR = 1.17, 95% CI = 1.06–1.30, *p* = 0.002), depression (aHR = 1.96, 95% CI = 1.75–2.20, *p* < 0.001), anxiety (aHR = 1.17, 95% CI = 1.06–1.29, *p* = 0.002), alcoholism (aHR = 2.44, 95% CI = 1.22–4.88, *p* = 0.012), and chronic obstructive pulmonary disease (aHR = 1.08, 95% CI = 1.02–1.14, *p* = 0.010). Moreover, the dementia-free survival rates differed between the three distinct trajectory groups, as estimated by the Kaplan–Meier method (*p* < 0.001), with the lowest survival rate in the high group, followed by the moderate and low groups (all pairwise comparisons: *p* < 0.001; Figure 2).

## 4. Discussion

During a 14-year period, this longitudinal study identified three distinct developmental trajectories of incident dementia, using a nationwide representative sample. Those diagnosed with hypertension, stroke, coronary heart disease, heart failure, and chronic obstructive pulmonary disease at baseline tended to be classified into the high- and moderate-incidence groups rather than the low-incidence group in dementia risk. Such factors were more critical than traditional risk factors, as shown in the sensitivity analysis, which identified elderly patients, women, and those with diabetes mellitus, stroke, coronary heart disease, kidney disease, depression, anxiety, alcoholism, and chronic obstructive pulmonary disease as having a higher risk of developing dementia. Moreover, the lowest dementia-free survival rates were found in high-incidence groups, followed by the moderate- and low-incidence groups.

In this study, the patients were clustered into three distinct dementia trajectories: high, moderate, and low. However, these findings contradict those of previous studies as their trajectories presented completely different fluctuation trends [12,13,14,15]. Some studies have shown distinctive trajectories without crossover [13,14], whereas others have reported overlapping trajectories of dementia [12,15]. A potential reason for the discrepancies among these studies might be the different definitions of dementia, such as the Clinical Dementia Rating Scale, Mini-Mental State Examination, 30-mintute delayed recall score, or composite assessments. In addition, previous studies have mostly investigated dynamic changes in cognitive functions over time among elderly participants. In contrast, our study attempted to classify the temporal trends of incident dementia by using a cohort study design. This may be another explanation for the variation between studies.

Regarding comparisons between the three distinct trajectory groups, our study found that those diagnosed with hypertension, stroke, coronary heart disease, heart failure, and chronic obstructive pulmonary disease had increased odds for being classified into the high- and moderate-incidence groups. The findings of the current study are comparable to those of previous studies [22,23,24,25]. These cardiovascular diseases may cause physical disability and cognitive impairment in older adults [22]. Moreover, accelerated cognitive decline and progression of dementia might be independently enhanced by cardiometabolic multimorbidity [23,24,25]. In addition, chronic obstructive pulmonary disease can be used as a surrogate marker for smoking behavior [26], which also has an impact on cognitive functioning [27]. However, the results of our study were slightly different when performing sensitivity analyses among the entire study cohort. Old age, female sex, diabetes mellitus, stroke, coronary heart disease, kidney disease, depression, anxiety, alcoholism, and chronic obstructive pulmonary disease were predictors of dementia. This may be because patients with comorbidities might be predisposed to severe deterioration in cognitive performance [28], whereas age is the most common cause of dementia [29].

Multimorbidity is prevalent in the elderly population, particularly cardiovascular diseases co-occurring with cognitive deficits [30,31]. However, the pathogenesis of these comorbidities remains unclear, although it is possible that cardiovascular disease and cognition may share several biological pathways [31]. Cardiovascular diseases may induce vascular pathogenesis, structural changes in the brain, inflammation, immunomodulation, and endocrine and metabolic disorders. These negative effects could subsequently aggravate cognitive impairment through neurodegeneration [32], reduced brain-derived neurotrophic factor generation [33], and the deposition of amyloid beta plaques [34]. Therefore, the onset of dementia can be delayed by effective interventional strategies to promote cardiovascular health [35].

This study had several strengths. First, the data source was the NHIRD, which consists of a nationwide sample and covers a 14-year longitudinal period. It presents considerable representativeness by extrapolating its validity to the entire Taiwanese population. Second, the patients could be clustered into different groups based on significant variations in the incidence of dementia by GBTM, but not the sample characteristics [36]. Therefore, the extension of GBTM could be used to estimate the predictors of dementia among three distinct trajectory groups. Third, the predictors and incidence of dementia were defined according to diagnostic codes in the NHIRD. Thus, diagnosis accuracy is guaranteed, which reduces the probability of misclassification bias.

In addition, the current study has some major limitations. First, because the data were obtained from claims data, information regarding the severity of dementia is lacking. To a certain degree, patients with mild cognitive impairment might not have been stratified and further investigated in this study. Second, detailed laboratory results and lifestyle factors are not available in the NHIRD database. Therefore, the extrapolation of findings regarding the predictors of dementia trajectories might be limited. Third, the independent and dependent variables in this study both have a long latency period. Therefore, it is difficult to determine causality between the predictors and outcomes.

## 5. Conclusions

In conclusion, the results of this 14-year longitudinal study identified three distinct trajectories of incident dementia among elderly Taiwanese people: high, moderate, and low. Moreover, patients with cardiovascular disease risk factors and cardiovascular disease events tended to be classified into the high- and moderate-incidence dementia groups. In addition, a sensitivity analysis for the entire sample revealed an increased risk of dementia in patients with advanced age, female sex, diabetes mellitus, stroke, coronary heart disease, kidney disease, depression, anxiety, alcoholism, and chronic obstructive pulmonary disease. Moreover, the three distinct trajectory groups significantly differed, with the lowest dementia-free survival rate observed in the high-incidence group. Early detection and management of these associated risk factors in the elderly may prevent or delay the deterioration of cognitive decline.

## Figures and Tables

**Table 1:** Distribution of sample characteristics according to dementia trajectory groups.

		Dementia Incidence by Trajectory Groups			*p* Value ^a^
	Total (*n* = 42,407)	High (*n* = 11,637)	Moderate (*n* = 19,036)	Low (*n* = 11,734)	
Age, mean (s.d.)	70.99 (4.76)	77.25 (3.31)	70.17 (1.94)	66.1 (1.05)	<0.001
Sex, n (%)					<0.001
Man	20,726 (48.87)	4578 (39.3)	8880 (46.6)	7268 (61.9)	
Women	21,681 (51.13)	7059 (60.7)	10,156 (53.4)	4466 (38.1)	
Diabetes mellitus, n (%)					<0.001
Yes	5441 (12.83)	1439 (12.4)	2608 (13.7)	1394 (11.9)	
No	36,966 (87.17)	10,198 (87.6)	16,428 (86.3)	10,340 (88.1)	
Hypertension, n (%)					<0.001
Yes	15,105 (35.62)	4644 (39.9)	6955 (36.5)	3506 (29.9)	
No	27,302 (64.38)	6993 (60.1)	12,081 (63.5)	8228 (70.1)	
Hyperlipidemia, n (%)					0.012
Yes	4579 (10.8)	1197 (10.3)	2148 (11.3)	1234 (10.5)	
No	37,828 (89.2)	10,440 (89.7)	16,888 (88.7)	10,500 (89.5)	
Stroke, n (%)					<0.001
Yes	3373 (7.95)	1172 (10.1)	1478 (7.8)	723 (6.2)	
No	39,034 (92.05)	10,465 (89.9)	17,558 (92.2)	11,011 (93.8)	
Coronary heart disease, n (%)					<0.001
Yes	6065 (14.3)	1958 (16.8)	2766 (14.5)	1341 (11.4)	
No	36,342 (85.7)	9679 (83.2)	16,270 (85.5)	10,393 (88.6)	
Kidney disease, n (%)					0.004
Yes	1624 (3.83)	465 (4)	768 (4)	391 (3.3)	
No	40,783 (96.17)	11,172 (96)	18,268 (96)	11,343 (96.7)	
Atrial fibrillation, n (%)					0.005
Yes	349 (0.82)	122 (1)	146 (0.8)	81 (0.7)	
No	42,058 (99.18)	11,515 (99)	18,890 (99.2)	11,653 (99.3)	
Depression, n (%)					0.387
Yes	835 (1.97)	246 (2.1)	369 (1.9)	220 (1.9)	
No	41,572 (98.03)	11,391 (97.9)	18,667 (98.1)	11,514 (98.1)	
Anxiety, n (%)					0.009
Yes	1568 (3.7)	465 (4)	720 (3.8)	383 (3.3)	
No	40,839 (96.3)	11,172 (96)	18,316 (96.2)	11,351 (96.7)	
Heart failure, n (%)					<0.001
Yes	1078 (2.54)	412 (3.5)	462 (2.4)	204 (1.7)	
No	41,329 (97.46)	11,225 (96.5)	18,574 (97.6)	11,530 (98.3)	
Alcoholism, n (%)					0.462
Yes	19 (0.04)	3 (0)	9 (0)	7 (0.1)	
No	42,388 (99.96)	11,634 (100)	19,027 (100)	11,727 (99.9)	
COPD, n (%)					<0.001
Yes	6882 (16.23)	2017 (17.3)	3178 (16.7)	1687 (14.4)	
No	35,525 (83.77)	9620 (82.7)	15,858 (83.3)	10,047 (85.6)	
Obesity, n (%)					0.024
Yes	41 (0.1)	8 (0.1)	27 (0.1)	6 (0.1)	
No	42,366 (99.9)	11,629 (99.9)	19,009 (99.9)	11,728 (99.9)	

S.D., standard deviation; COPD, chronic obstructive pulmonary disease. ^a^ Tested by Kruskal–Wallis H test and chi-square test.

**Table 2:** Baseline characteristics associated with dementia trajectory groups.

Baseline Characteristics	High Group vs. Low Group			Moderate Group vs. Low Group		
	OR	95% CI	*p* Value ^a^	OR	95% CI	*p* Value ^a^
Diabetes mellitus	0.93	0.86–1.01	0.086	1.08	1.00–1.16	0.039
Hypertension	1.43	1.35–1.52	<0.001	1.27	1.20–1.34	<0.001
Hyperlipidemia	0.81	0.74–0.89	<0.001	0.94	0.86–1.01	0.097
Stroke	1.45	1.31–1.60	<0.001	1.14	1.03–1.25	0.008
Coronary heart disease	1.29	1.19–1.39	<0.001	1.16	1.07–1.24	<0.001
Kidney disease	1.07	0.93–1.23	0.349	1.11	0.98–1.26	0.103
Atrial fibrillation	1.07	0.80–1.43	0.653	0.92	0.70–1.21	0.552
Depression	0.97	0.81–1.17	0.756	0.95	0.80–1.12	0.526
Anxiety	1.05	0.92–1.21	0.461	1.06	0.93–1.20	0.380
Heart failure	1.62	1.36–1.93	<0.001	1.19	1.00–1.41	0.048
Alcoholism	0.32	0.08–1.27	0.105	0.66	0.25–1.79	0.416
COPD	1.10	1.02–1.18	0.009	1.11	1.04–1.19	0.002
Obesity	1.23	0.42–3.56	0.705	2.50	1.03–6.06	0.043

Reference: Low dementia incidence group. OR, odds ratio; CI, confidence interval; COPD, chronic obstructive pulmonary disease. ^a^ Tested using multinomial logistic regression.

**Table 3:** Baseline characteristics predicted the risk of dementia for the whole sample.

Baseline Characteristics	HR	95% CI	*p* Value ^a^
Age	1.08	1.08–1.09	<0.001
Sex (Female)	1.20	1.15–1.25	<0.001
Diabetes mellitus	1.26	1.18–1.34	<0.001
Hypertension	1.01	0.96–1.06	0.668
Hyperlipidemia	1.00	0.94–1.07	0.950
Stroke	1.61	1.51–1.72	<0.001
CHD	1.13	1.06–1.20	<0.001
Kidney disease	1.17	1.06–1.30	0.002
Atrial fibrillation	1.05	0.85–1.30	0.671
Depression	1.96	1.75–2.20	<0.001
Anxiety	1.17	1.06–1.29	0.002
Heart failure	0.94	0.83–1.07	0.362
Alcoholism	2.44	1.22–4.88	0.012
COPD	1.08	1.02–1.14	0.010
Obesity	1.55	0.88–2.74	0.127

HR, hazard ratio; CI, confidence interval; CHD, coronary heart disease; COPD, chronic obstructive pulmonary disease. ^a^ Tested using multivariate Cox proportional hazard regression.

## Data Availability

Data described in the manuscript, code book, and analytic code will not be made available because the data source used in this study was managed by the National Health Research Institutes, which researchers need to apply to for scientific purposes, and these data were not publicly accessible.
